# Carbonyl­chlorido(1-methyl­sulfanylpenta-1,3-dien-1-yl-5-yl­idene)bis­(triphenyl­phosphane)osmium(II)

**DOI:** 10.1107/S1600536809039695

**Published:** 2009-10-07

**Authors:** Paul M. Johns, Warren R. Roper, Scott D. Woodgate, L. James Wright

**Affiliations:** a Department of Chemistry, The University of Auckland, Private Bag 92019, Auckland, New Zealand

## Abstract

The crystal structure of the title compound, [Os(C_6_H_7_S)Cl(C_18_H_15_P)_2_(CO)], confirms the formulation as an osmabenzene. There is a slightly distorted octa­hedral coordination environment at the Os^II^ ion, with the triphenyl­phosphane ligands mutually *trans* and the chloride *cis* to the carbon bearing the –SMe substituent. Within the metallacyclic ring, the C—C distances are appropriate for aromatic bonds and the two Os—C distances are shorter than typical Os—C single bonds. The maximum deviation from the least-squares plane through the osmabenzene ring occurs for the carbon bearing the SMe substituent [0.1037 (18) Å].

## Related literature

For the synthesis and properties of metallabenzenes, see: Bleeke (2001 ▶); Landorf & Haley (2006 ▶); Wright (2006 ▶). For the synthesis and properties of osmabenzenes, see: Elliott *et al.* (1982 ▶, 1989 ▶); Rickard *et al.* (2000 ▶, 2001 ▶). For a discussion of ring planarity in metallabenzenes, see: Zhu *et al.* (2007 ▶). For spectroscopic data, see: Maddock *et al.* (1996 ▶).
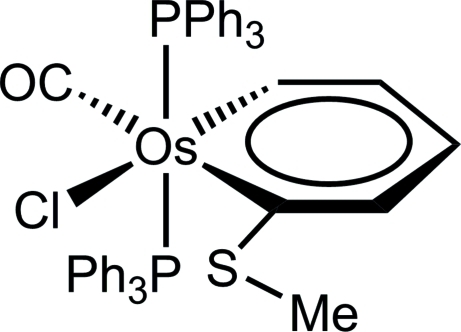


## Experimental

### 

#### Crystal data

[Os(C_6_H_7_S)Cl(C_18_H_15_P)_2_(CO)]
*M* *_r_* = 889.38
Monoclinic, 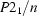

*a* = 13.5565 (1) Å
*b* = 15.7136 (2) Å
*c* = 18.2264 (3) Å
β = 109.978 (1)°
*V* = 3648.97 (8) Å^3^
*Z* = 4
Mo *K*α radiation
μ = 3.75 mm^−1^
*T* = 203 K
0.33 × 0.28 × 0.11 mm

#### Data collection

Siemens SMART CCD area-detector diffractometer
Absorption correction: multi-scan (*SADABS*; Sheldrick, 1996 ▶) *T*_min_ = 0.491, *T*_max_ = 0.739
22209 measured reflections
7812 independent reflections
6032 reflections with *I* > 2σ(*I*)
*R*_int_ = 0.023

#### Refinement

*R*[*F* ^2^ > 2σ(*F* ^2^)] = 0.022
*wR*(*F* ^2^) = 0.060
*S* = 1.01
7812 reflections
444 parameters
H-atom parameters constrained
Δρ_max_ = 1.01 e Å^−3^
Δρ_min_ = −0.53 e Å^−3^

### 

Data collection: *SMART* (Siemens, 1995 ▶); cell refinement: *SAINT* (Siemens, 1995 ▶); data reduction: *SAINT*; program(s) used to solve structure: *SHELXS97* (Sheldrick, 2008 ▶); program(s) used to refine structure: *SHELXL97* (Sheldrick, 2008 ▶); molecular graphics: *ORTEP-3 for Windows* (Farrugia, 1997 ▶); software used to prepare material for publication: *WinGX* (Farrugia, 1999 ▶).

## Supplementary Material

Crystal structure: contains datablocks I, global. DOI: 10.1107/S1600536809039695/lh2916sup1.cif

Structure factors: contains datablocks I. DOI: 10.1107/S1600536809039695/lh2916Isup2.hkl

Additional supplementary materials:  crystallographic information; 3D view; checkCIF report

## Figures and Tables

**Table 1:** Selected bond lengths (Å)

Os1—C1	2.109 (3)
Os1—C5	2.026 (3)
C1—C2	1.410 (4)
C2—C3	1.370 (4)
C3—C4	1.393 (5)
C4—C5	1.367 (4)

**Fig. 1.**
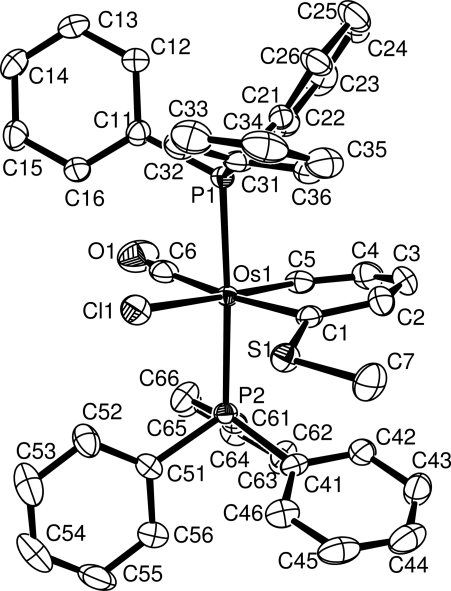
The molecular structure of [Os(C5H4{SMe–1})Cl(CO)(PPh3)2] showing 50% probability displacement ellipsoids for non-hydrogen atoms. H atoms omitted for clarity.

## supplementary crystallographic information

### Comment

Metallabenzenes are now a well established class of organometallic compounds and a considerable number of studies involving the syntheses, reactivity and aromatic character of these materials have been made. To obtain further data relating to the nature of the delocalized bonding in osmabenzenes (Rickard *et al.*, 2000; Rickard *et al.*, 2001) we have obtained the single-crystal *X*–ray structure of the title complex [Os(C_5_H_4_{SMe–1})Cl(CO)(PPh_3_)_2_]. The geometry about Os is approximately octahedral with the two PPh_3_ ligands mutually *trans*. Within the metallacyclic ring the Os—C1 and Os—C5 distances are shorter than those observed for normal Os—C single bonds suggesting there is some multiple character to these bonds (see Table 1). The C—C distances in this ring are very close to those found in simple aromatic compounds and come within the range of distances reported for other metallabenzenes (Bleeke, 2001; Landorf & Haley, 2006; Wright, 2006). The osmabenzene ring is not planar and the atoms that show the greatest displacement from the mean plane through Os and the five ring carbons are C1 (0.1037 (18) Å) and Os (0.1000 (13) Å). Non-planarity has been observed for a number of other metallabenzenes. This phenomenon has been investigated theoretically and shown not to compromise the electron delocalization within the ring (Zhu *et al.*, 2007).

### Experimental

[Os(C_5_H_4_{S–1})(CO)(PPh_3_)_2_] (Elliott *et al.*, 1982; Elliott *et al.*, 1989) (200 mg, 0.238 mmol) was dissolved in dry dichloromethane (25 ml) and methyl trifluoromethanesulfonate (534 µ*L*, 0.48 mmol) was added. NaCl (27.8 mg, 0.476 mmol) dissolved in water (1 ml) was added to the blue solution and the mixture stirred for one hour. The dichloromethane layer was seperated and then eluted through a chromatography column (silica gel support, 2.5 cm *x* 1.5 cm) using dichloromethane as the eluent. The fast-moving dark blue band was collected and recrystallized from dichloromethane/ethanol (25 ml/10 ml) to give crystals of the title compound (188 mg, 89%). The crystal used for the single-crystal X-ray diffraction study was also grown from dichloromethane/ethanol. MS: Calcd for C_43_H_37_OOsP_2_S [M–Cl]^+^ 853.1624. Found: 853.1603 *m/z*. Anal. Found: C, 57.50; H 4.19. C_43_H_37_ClOOsP_2_S requires C, 58.07; H, 4.19%. ^1^H NMR (CDCl_3_, δ): 1.70 (s, 3H, SC*H*_3_), 6.57 (apparent t, 1H, *H4*, ^3^*J*_HH_ = 8.5 Hz), 6.65 (d, 1H, *H2*, ^3^*J*_HH_ = 8.8 Hz), 7.07 (d apparent t, 1H, *H3*, ^3^*J*_HH_ = 8.8 Hz, ^4^*J*_HH_ = 1.7 Hz), 7.45–7.69 (m, 30H, P*Ph*_3_), 13.27 (d, 1H, *H5*, ^3^*J*_HH_ = 9.3 Hz). ^13^C{^1^H} NMR (CDCl_3_, δ): 23.21 (s, S*C*H_3_), 121.60 (s, *C2*), 123.75 (s, *C4*), 126.89 (t'(Maddock *et al.*, 1996), *o*-P*Ph_3_*, ^2,4^*J*_CP_ = 10.1 Hz), 129.32 (s, *p*-P*Ph_3_*), 133.22 (t', *i*-P*Ph_3_*, ^1,3^*J*_CP_ = 53.3 Hz), 134.62 (t', *m*-P*Ph_3_*, ^3,5^*J*_CP_ = 11.1 Hz), 145.81 (s, *C3*), 191.61 (t, *C*O, ^2^*J*_CP_ = 11.1 Hz), 220.96 (t, *C5*, ^2^*J*_CP_ = 6.3 Hz), 237.42 (t, *C1*, ^2^*J*_CP_ = 9.1 Hz).

### Refinement

Hydrogen atoms were placed in calculated positions and refined using the riding model [C— H 0.93–0.97 Å, with *U*_iso_(H) = 1.2 or 1.5 times *U*_eq_(C). The highest density peak and deepest hole are located 0.84 Å and 0.54 Å from atoms Os1 and Cl1 respectively.

### Crystal data

**Table:**

[Os(C_6_H_7_S)Cl(C_18_H_15_P)_2_(CO)]	*F*(000) = 1768
*M**_r_* = 889.38	*D*_x_ = 1.619 Mg m^−^^3^
Monoclinic, *P*2_1_/*n*	Mo *K*α radiation, λ = 0.71073 Å
Hall symbol: -P 2yn	Cell parameters from 8192 reflections
*a* = 13.5565 (1) Å	θ = 1.6–28.3°
*b* = 15.7136 (2) Å	µ = 3.75 mm^−^^1^
*c* = 18.2264 (3) Å	*T* = 203 K
β = 109.978 (1)°	Plates, blue
*V* = 3648.97 (8) Å^3^	0.33 × 0.28 × 0.11 mm
*Z* = 4	

### Data collection

**Table:**

Siemens SMART CCD area-detector diffractometer	7812 independent reflections
Radiation source: fine-focus sealed tube	6032 reflections with *I* > 2σ(*I*)
graphite	*R*_int_ = 0.023
ω scans	θ_max_ = 27.0°, θ_min_ = 1.6°
Absorption correction: multi-scan (*SADABS*; Sheldrick, 1996)	*h* = −17→16
*T*_min_ = 0.491, *T*_max_ = 0.739	*k* = 0→19
22209 measured reflections	*l* = 0→23

### Refinement

**Table:**

Refinement on *F*^2^	Secondary atom site location: difference Fourier map
Least-squares matrix: full	Hydrogen site location: inferred from neighbouring sites
*R*[*F*^2^ > 2σ(*F*^2^)] = 0.022	H-atom parameters constrained
*wR*(*F*^2^) = 0.060	*w* = 1/[σ^2^(*F*_o_^2^) + (0.0268*P*)^2^ + 2.9139*P*] where *P* = (*F*_o_^2^ + 2*F*_c_^2^)/3
*S* = 1.01	(Δ/σ)_max_ = 0.004
7812 reflections	Δρ_max_ = 1.01 e Å^−^^3^
444 parameters	Δρ_min_ = −0.53 e Å^−^^3^
0 restraints	Extinction correction: *SHELXL97* (Sheldrick, 2008), Fc^*^=kFc[1+0.001xFc^2^λ^3^/sin(2θ)]^-1/4^
Primary atom site location: structure-invariant direct methods	Extinction coefficient: 0.00065 (5)

### Special details

**Table:**

Geometry. All e.s.d.'s (except the e.s.d. in the dihedral angle between two l.s. planes) are estimated using the full covariance matrix. The cell e.s.d.'s are taken into account individually in the estimation of e.s.d.'s in distances, angles and torsion angles; correlations between e.s.d.'s in cell parameters are only used when they are defined by crystal symmetry. An approximate (isotropic) treatment of cell e.s.d.'s is used for estimating e.s.d.'s involving l.s. planes.
Refinement. Refinement of *F^2^* against ALL reflections. The weighted *R*-factor *wR* and goodness of fit *S* are based on *F^2^*, conventional *R*-factors *R* are based on *F*, with *F* set to zero for negative *F^2^*. The threshold expression of *F^2^* > 2σ(*F^2^*) is used only for calculating *R*-factors(gt) *etc*. and is not relevant to the choice of reflections for refinement. *R*-factors based on *F^2^* are statistically about twice as large as those based on *F*, and *R*- factors based on ALL data will be even larger.

### Fractional atomic coordinates and isotropic or equivalent isotropic displacement parameters (Å^2^)

**Table:**

	*x*	*y*	*z*	*U*_iso_*/*U*_eq_	
Os1	0.503721 (7)	0.750195 (6)	0.123794 (5)	0.01728 (5)	
Cl1	0.52137 (6)	0.89985 (5)	0.17787 (4)	0.03293 (16)	
P1	0.35379 (5)	0.75535 (4)	0.16571 (4)	0.02055 (14)	
P2	0.65608 (5)	0.75710 (4)	0.08534 (4)	0.02050 (14)	
S1	0.41485 (6)	0.90387 (5)	0.00041 (4)	0.02726 (15)	
O1	0.6180 (2)	0.6574 (2)	0.26956 (18)	0.0572 (7)	
C1	0.4083 (2)	0.79638 (18)	0.01381 (16)	0.0235 (6)	
C2	0.3413 (2)	0.74700 (19)	−0.04778 (18)	0.0314 (7)	
H2A	0.2937	0.7754	−0.0907	0.038*	
C3	0.3422 (2)	0.6598 (2)	−0.04821 (18)	0.0351 (7)	
H3A	0.2913	0.6336	−0.0909	0.042*	
C4	0.4089 (3)	0.6051 (2)	0.00668 (19)	0.0353 (7)	
H4A	0.4057	0.5466	−0.0047	0.042*	
C5	0.4789 (2)	0.63212 (18)	0.07632 (18)	0.0280 (6)	
H5A	0.5222	0.5893	0.1069	0.034*	
C6	0.5838 (2)	0.6926 (2)	0.2226 (2)	0.0331 (7)	
C7	0.3395 (3)	0.9277 (2)	−0.10003 (18)	0.0413 (8)	
H7A	0.3550	0.9851	−0.1122	0.062*	
H7B	0.2652	0.9229	−0.1079	0.062*	
H7C	0.3577	0.8878	−0.1340	0.062*	
C11	0.3842 (2)	0.76453 (17)	0.27235 (16)	0.0235 (6)	
C12	0.3095 (3)	0.7412 (2)	0.30511 (19)	0.0353 (7)	
H12A	0.2436	0.7214	0.2727	0.042*	
C13	0.3310 (3)	0.7467 (2)	0.3850 (2)	0.0426 (9)	
H13A	0.2797	0.7303	0.4063	0.051*	
C14	0.4268 (3)	0.7760 (2)	0.43332 (18)	0.0374 (8)	
H14A	0.4411	0.7795	0.4875	0.045*	
C15	0.5017 (2)	0.8001 (2)	0.40201 (17)	0.0326 (7)	
H15A	0.5673	0.8203	0.4347	0.039*	
C16	0.4802 (2)	0.79462 (19)	0.32186 (16)	0.0278 (6)	
H16A	0.5316	0.8115	0.3008	0.033*	
C21	0.2689 (2)	0.6613 (2)	0.14554 (17)	0.0290 (7)	
C22	0.3159 (3)	0.5816 (2)	0.16040 (18)	0.0359 (7)	
H22A	0.3894	0.5775	0.1759	0.043*	
C23	0.2570 (3)	0.5082 (2)	0.1529 (2)	0.0493 (10)	
H23A	0.2900	0.4547	0.1634	0.059*	
C24	0.1479 (3)	0.5146 (3)	0.1294 (2)	0.0570 (12)	
H24A	0.1070	0.4652	0.1243	0.068*	
C25	0.1005 (3)	0.5922 (3)	0.1140 (2)	0.0549 (11)	
H25A	0.0270	0.5958	0.0976	0.066*	
C26	0.1597 (3)	0.6659 (2)	0.12214 (19)	0.0406 (8)	
H26A	0.1262	0.7192	0.1119	0.049*	
C31	0.2631 (2)	0.8443 (2)	0.12691 (16)	0.0253 (6)	
C32	0.2611 (2)	0.9141 (2)	0.17292 (19)	0.0314 (7)	
H32A	0.3027	0.9141	0.2261	0.038*	
C33	0.1988 (3)	0.9836 (2)	0.1415 (2)	0.0401 (8)	
H33A	0.1983	1.0304	0.1734	0.048*	
C34	0.1374 (2)	0.9848 (2)	0.0637 (2)	0.0428 (9)	
H34A	0.0958	1.0326	0.0424	0.051*	
C35	0.1373 (2)	0.9154 (2)	0.0172 (2)	0.0409 (8)	
H35A	0.0947	0.9157	−0.0357	0.049*	
C36	0.1997 (2)	0.8454 (2)	0.04829 (18)	0.0335 (7)	
H36A	0.1994	0.7984	0.0163	0.040*	
C41	0.6232 (2)	0.78681 (19)	−0.01726 (16)	0.0249 (6)	
C42	0.5683 (2)	0.7288 (2)	−0.07572 (18)	0.0325 (7)	
H42A	0.5528	0.6740	−0.0622	0.039*	
C43	0.5370 (3)	0.7532 (2)	−0.1540 (2)	0.0442 (9)	
H43A	0.5027	0.7138	−0.1932	0.053*	
C44	0.5558 (3)	0.8340 (3)	−0.1742 (2)	0.0459 (9)	
H44A	0.5347	0.8497	−0.2271	0.055*	
C45	0.6055 (3)	0.8925 (2)	−0.1170 (2)	0.0426 (9)	
H45A	0.6161	0.9485	−0.1309	0.051*	
C46	0.6399 (2)	0.8687 (2)	−0.03906 (19)	0.0321 (7)	
H46A	0.6749	0.9085	−0.0005	0.038*	
C51	0.7660 (2)	0.82842 (19)	0.13641 (17)	0.0265 (6)	
C52	0.7716 (2)	0.8719 (2)	0.2035 (2)	0.0363 (8)	
H52A	0.7183	0.8653	0.2252	0.044*	
C53	0.8567 (3)	0.9258 (2)	0.2393 (2)	0.0502 (10)	
H53A	0.8599	0.9559	0.2846	0.060*	
C54	0.9353 (3)	0.9348 (2)	0.2086 (2)	0.0512 (10)	
H54A	0.9913	0.9723	0.2321	0.061*	
C55	0.9324 (3)	0.8887 (3)	0.1431 (2)	0.0523 (10)	
H55A	0.9878	0.8931	0.1233	0.063*	
C56	0.8481 (3)	0.8365 (2)	0.1071 (2)	0.0413 (8)	
H56A	0.8458	0.8060	0.0623	0.050*	
C61	0.7313 (2)	0.65751 (18)	0.09802 (17)	0.0233 (6)	
C62	0.7491 (2)	0.6108 (2)	0.03920 (18)	0.0320 (7)	
H62A	0.7202	0.6292	−0.0129	0.038*	
C63	0.8093 (3)	0.5370 (2)	0.0568 (2)	0.0389 (8)	
H63A	0.8204	0.5057	0.0164	0.047*	
C64	0.8526 (2)	0.5094 (2)	0.13256 (19)	0.0334 (7)	
H64A	0.8927	0.4592	0.1440	0.040*	
C65	0.8371 (3)	0.5558 (2)	0.19159 (19)	0.0364 (7)	
H65A	0.8668	0.5375	0.2436	0.044*	
C66	0.7779 (2)	0.6290 (2)	0.17438 (18)	0.0353 (7)	
H66A	0.7688	0.6606	0.2153	0.042*	

### Atomic displacement parameters (Å^2^)

**Table:**

	*U*^11^	*U*^22^	*U*^33^	*U*^12^	*U*^13^	*U*^23^
Os1	0.01787 (6)	0.01638 (6)	0.01841 (6)	−0.00104 (4)	0.00725 (4)	0.00086 (4)
Cl1	0.0314 (4)	0.0327 (4)	0.0342 (4)	−0.0012 (3)	0.0105 (3)	0.0008 (3)
P1	0.0185 (3)	0.0244 (4)	0.0191 (3)	−0.0022 (3)	0.0069 (3)	0.0012 (3)
P2	0.0199 (3)	0.0218 (4)	0.0214 (3)	−0.0007 (3)	0.0091 (3)	0.0001 (3)
S1	0.0287 (4)	0.0259 (4)	0.0262 (4)	0.0020 (3)	0.0081 (3)	0.0052 (3)
O1	0.0490 (16)	0.068 (2)	0.0625 (19)	0.0120 (15)	0.0288 (15)	0.0092 (16)
C1	0.0216 (13)	0.0274 (15)	0.0245 (14)	−0.0001 (11)	0.0117 (11)	0.0008 (12)
C2	0.0269 (14)	0.0362 (17)	0.0276 (15)	−0.0031 (13)	0.0048 (12)	−0.0002 (13)
C3	0.0302 (16)	0.0405 (19)	0.0320 (17)	−0.0140 (14)	0.0072 (13)	−0.0079 (14)
C4	0.0419 (18)	0.0235 (15)	0.0453 (19)	−0.0079 (14)	0.0210 (15)	−0.0047 (14)
C5	0.0300 (15)	0.0228 (14)	0.0366 (17)	−0.0020 (12)	0.0187 (13)	−0.0002 (12)
C6	0.0261 (16)	0.0375 (19)	0.0424 (19)	−0.0048 (14)	0.0207 (15)	−0.0094 (15)
C7	0.049 (2)	0.044 (2)	0.0268 (16)	0.0060 (16)	0.0071 (15)	0.0135 (14)
C11	0.0260 (14)	0.0235 (14)	0.0205 (13)	−0.0003 (11)	0.0073 (11)	0.0017 (11)
C12	0.0294 (15)	0.051 (2)	0.0290 (16)	−0.0121 (14)	0.0145 (13)	−0.0062 (14)
C13	0.0410 (18)	0.064 (3)	0.0313 (17)	−0.0146 (17)	0.0228 (15)	−0.0024 (16)
C14	0.0458 (19)	0.0481 (19)	0.0208 (15)	−0.0048 (16)	0.0143 (14)	−0.0001 (14)
C15	0.0336 (17)	0.0376 (18)	0.0224 (15)	−0.0039 (13)	0.0041 (13)	0.0000 (13)
C16	0.0281 (15)	0.0322 (16)	0.0240 (14)	−0.0053 (13)	0.0101 (12)	0.0019 (12)
C21	0.0314 (15)	0.0355 (17)	0.0206 (14)	−0.0126 (13)	0.0096 (12)	−0.0002 (12)
C22	0.0433 (19)	0.0350 (18)	0.0321 (17)	−0.0121 (15)	0.0166 (15)	0.0006 (14)
C23	0.079 (3)	0.0332 (19)	0.040 (2)	−0.0201 (19)	0.0262 (19)	0.0002 (16)
C24	0.070 (3)	0.061 (3)	0.037 (2)	−0.043 (2)	0.0148 (19)	−0.0004 (19)
C25	0.0381 (19)	0.078 (3)	0.043 (2)	−0.030 (2)	0.0065 (16)	0.006 (2)
C26	0.0288 (16)	0.052 (2)	0.0386 (19)	−0.0146 (15)	0.0088 (14)	0.0055 (16)
C31	0.0167 (12)	0.0344 (17)	0.0274 (15)	0.0017 (12)	0.0107 (11)	0.0069 (12)
C32	0.0275 (15)	0.0335 (17)	0.0340 (16)	0.0028 (13)	0.0116 (13)	0.0033 (14)
C33	0.0327 (17)	0.0363 (19)	0.054 (2)	0.0054 (14)	0.0179 (16)	0.0041 (16)
C34	0.0255 (16)	0.043 (2)	0.062 (2)	0.0101 (14)	0.0178 (16)	0.0202 (18)
C35	0.0217 (15)	0.064 (2)	0.0350 (18)	0.0087 (15)	0.0067 (13)	0.0186 (17)
C36	0.0256 (15)	0.0463 (19)	0.0299 (16)	0.0022 (14)	0.0110 (13)	0.0041 (14)
C41	0.0243 (14)	0.0291 (15)	0.0246 (14)	0.0035 (12)	0.0127 (12)	0.0050 (12)
C42	0.0258 (15)	0.0433 (18)	0.0278 (16)	−0.0019 (13)	0.0084 (13)	0.0027 (13)
C43	0.0329 (17)	0.071 (3)	0.0270 (16)	−0.0006 (17)	0.0081 (14)	−0.0040 (17)
C44	0.0354 (18)	0.073 (3)	0.0328 (18)	0.0145 (18)	0.0162 (15)	0.0186 (18)
C45	0.0390 (18)	0.048 (2)	0.049 (2)	0.0121 (16)	0.0245 (17)	0.0231 (17)
C46	0.0315 (16)	0.0301 (16)	0.0377 (17)	0.0078 (13)	0.0158 (14)	0.0064 (13)
C51	0.0216 (13)	0.0247 (15)	0.0318 (16)	−0.0023 (11)	0.0072 (12)	0.0013 (12)
C52	0.0231 (15)	0.0390 (19)	0.0425 (19)	0.0009 (13)	0.0057 (14)	−0.0112 (15)
C53	0.0356 (18)	0.045 (2)	0.058 (2)	0.0001 (16)	0.0003 (17)	−0.0200 (18)
C54	0.0326 (18)	0.037 (2)	0.072 (3)	−0.0124 (15)	0.0012 (18)	−0.0025 (19)
C55	0.0328 (18)	0.066 (3)	0.059 (2)	−0.0197 (18)	0.0167 (18)	0.008 (2)
C56	0.0372 (18)	0.051 (2)	0.0406 (19)	−0.0133 (16)	0.0191 (16)	−0.0053 (16)
C61	0.0217 (13)	0.0223 (14)	0.0284 (15)	0.0008 (11)	0.0117 (11)	0.0008 (12)
C62	0.0350 (16)	0.0359 (17)	0.0262 (15)	0.0035 (14)	0.0117 (13)	0.0004 (13)
C63	0.0455 (19)	0.0341 (18)	0.0397 (18)	0.0071 (15)	0.0177 (16)	−0.0108 (14)
C64	0.0316 (16)	0.0246 (15)	0.0435 (19)	0.0037 (13)	0.0120 (14)	0.0012 (14)
C65	0.0362 (17)	0.0371 (18)	0.0347 (17)	0.0111 (14)	0.0106 (14)	0.0067 (14)
C66	0.0381 (18)	0.0425 (19)	0.0258 (16)	0.0130 (14)	0.0118 (14)	0.0029 (14)

### Geometric parameters (Å, °)

**Table:**

Os1—C6	1.976 (4)	C25—H25A	0.9400
Os1—C1	2.109 (3)	C26—H26A	0.9400
Os1—C5	2.026 (3)	C31—C32	1.387 (4)
Os1—P2	2.3996 (7)	C31—C36	1.397 (4)
Os1—P1	2.4047 (7)	C32—C33	1.379 (4)
Os1—Cl1	2.5293 (8)	C32—H32A	0.9400
P1—C21	1.832 (3)	C33—C34	1.378 (5)
P1—C31	1.836 (3)	C33—H33A	0.9400
P1—C11	1.850 (3)	C34—C35	1.380 (5)
P2—C41	1.829 (3)	C34—H34A	0.9400
P2—C61	1.839 (3)	C35—C36	1.385 (4)
P2—C51	1.843 (3)	C35—H35A	0.9400
S1—C1	1.713 (3)	C36—H36A	0.9400
S1—C7	1.805 (3)	C41—C46	1.388 (4)
O1—C6	0.992 (4)	C41—C42	1.406 (4)
C1—C2	1.410 (4)	C42—C43	1.396 (5)
C2—C3	1.370 (4)	C42—H42A	0.9400
C2—H2A	0.9400	C43—C44	1.371 (5)
C3—C4	1.393 (5)	C43—H43A	0.9400
C3—H3A	0.9400	C44—C45	1.380 (5)
C4—C5	1.367 (4)	C44—H44A	0.9400
C4—H4A	0.9400	C45—C46	1.388 (4)
C5—H5A	0.9400	C45—H45A	0.9400
C7—H7A	0.9700	C46—H46A	0.9400
C7—H7B	0.9700	C51—C52	1.380 (4)
C7—H7C	0.9700	C51—C56	1.394 (4)
C11—C12	1.389 (4)	C52—C53	1.400 (4)
C11—C16	1.389 (4)	C52—H52A	0.9400
C12—C13	1.387 (4)	C53—C54	1.370 (6)
C12—H12A	0.9400	C53—H53A	0.9400
C13—C14	1.376 (5)	C54—C55	1.386 (6)
C13—H13A	0.9400	C54—H54A	0.9400
C14—C15	1.378 (4)	C55—C56	1.378 (5)
C14—H14A	0.9400	C55—H55A	0.9400
C15—C16	1.391 (4)	C56—H56A	0.9400
C15—H15A	0.9400	C61—C62	1.386 (4)
C16—H16A	0.9400	C61—C66	1.391 (4)
C21—C22	1.389 (5)	C62—C63	1.392 (4)
C21—C26	1.395 (4)	C62—H62A	0.9400
C22—C23	1.383 (4)	C63—C64	1.373 (5)
C22—H22A	0.9400	C63—H63A	0.9400
C23—C24	1.395 (6)	C64—C65	1.374 (4)
C23—H23A	0.9400	C64—H64A	0.9400
C24—C25	1.362 (6)	C65—C66	1.376 (4)
C24—H24A	0.9400	C65—H65A	0.9400
C25—C26	1.388 (5)	C66—H66A	0.9400
			
C6—Os1—C5	85.91 (13)	C23—C24—H24A	119.9
C6—Os1—C1	172.52 (12)	C24—C25—C26	120.7 (4)
C5—Os1—C1	87.18 (12)	C24—C25—H25A	119.7
C6—Os1—P2	91.54 (9)	C26—C25—H25A	119.7
C5—Os1—P2	87.16 (8)	C25—C26—C21	120.2 (4)
C1—Os1—P2	90.95 (7)	C25—C26—H26A	119.9
C6—Os1—P1	89.25 (9)	C21—C26—H26A	119.9
C5—Os1—P1	97.56 (8)	C32—C31—C36	118.5 (3)
C1—Os1—P1	88.84 (7)	C32—C31—P1	121.2 (2)
P2—Os1—P1	175.26 (2)	C36—C31—P1	120.1 (2)
C6—Os1—Cl1	97.00 (10)	C33—C32—C31	120.7 (3)
C5—Os1—Cl1	176.00 (9)	C33—C32—H32A	119.6
C1—Os1—Cl1	89.78 (8)	C31—C32—H32A	119.6
P2—Os1—Cl1	95.48 (2)	C34—C33—C32	120.5 (3)
P1—Os1—Cl1	79.78 (2)	C34—C33—H33A	119.7
C21—P1—C31	104.19 (15)	C32—C33—H33A	119.7
C21—P1—C11	99.96 (13)	C33—C34—C35	119.6 (3)
C31—P1—C11	103.00 (13)	C33—C34—H34A	120.2
C21—P1—Os1	116.76 (10)	C35—C34—H34A	120.2
C31—P1—Os1	115.50 (9)	C34—C35—C36	120.3 (3)
C11—P1—Os1	115.31 (10)	C34—C35—H35A	119.8
C41—P2—C61	106.08 (13)	C36—C35—H35A	119.8
C41—P2—C51	103.53 (14)	C35—C36—C31	120.3 (3)
C61—P2—C51	97.81 (13)	C35—C36—H36A	119.8
C41—P2—Os1	112.12 (9)	C31—C36—H36A	119.8
C61—P2—Os1	114.88 (9)	C46—C41—C42	118.7 (3)
C51—P2—Os1	120.54 (10)	C46—C41—P2	121.5 (2)
C1—S1—C7	108.06 (15)	C42—C41—P2	119.4 (2)
C2—C1—S1	118.7 (2)	C43—C42—C41	119.6 (3)
C2—C1—Os1	125.9 (2)	C43—C42—H42A	120.2
S1—C1—Os1	115.46 (15)	C41—C42—H42A	120.2
C3—C2—C1	123.4 (3)	C44—C43—C42	120.6 (3)
C3—C2—H2A	118.3	C44—C43—H43A	119.7
C1—C2—H2A	118.3	C42—C43—H43A	119.7
C2—C3—C4	128.2 (3)	C43—C44—C45	120.1 (3)
C2—C3—H3A	115.9	C43—C44—H44A	119.9
C4—C3—H3A	115.9	C45—C44—H44A	119.9
C5—C4—C3	123.3 (3)	C44—C45—C46	120.0 (3)
C5—C4—H4A	118.3	C44—C45—H45A	120.0
C3—C4—H4A	118.3	C46—C45—H45A	120.0
C4—C5—Os1	130.0 (2)	C45—C46—C41	120.9 (3)
C4—C5—H5A	115.0	C45—C46—H46A	119.6
Os1—C5—H5A	115.0	C41—C46—H46A	119.6
O1—C6—Os1	172.7 (4)	C52—C51—C56	119.0 (3)
S1—C7—H7A	109.5	C52—C51—P2	122.6 (2)
S1—C7—H7B	109.5	C56—C51—P2	118.4 (2)
H7A—C7—H7B	109.5	C51—C52—C53	119.9 (3)
S1—C7—H7C	109.5	C51—C52—H52A	120.0
H7A—C7—H7C	109.5	C53—C52—H52A	120.0
H7B—C7—H7C	109.5	C54—C53—C52	120.4 (4)
C12—C11—C16	118.0 (3)	C54—C53—H53A	119.8
C12—C11—P1	119.8 (2)	C52—C53—H53A	119.8
C16—C11—P1	122.1 (2)	C53—C54—C55	120.0 (3)
C13—C12—C11	120.8 (3)	C53—C54—H54A	120.0
C13—C12—H12A	119.6	C55—C54—H54A	120.0
C11—C12—H12A	119.6	C56—C55—C54	119.7 (3)
C14—C13—C12	120.4 (3)	C56—C55—H55A	120.1
C14—C13—H13A	119.8	C54—C55—H55A	120.1
C12—C13—H13A	119.8	C55—C56—C51	120.9 (3)
C13—C14—C15	119.7 (3)	C55—C56—H56A	119.5
C13—C14—H14A	120.1	C51—C56—H56A	119.5
C15—C14—H14A	120.1	C62—C61—C66	117.6 (3)
C14—C15—C16	119.9 (3)	C62—C61—P2	126.0 (2)
C14—C15—H15A	120.1	C66—C61—P2	116.3 (2)
C16—C15—H15A	120.1	C61—C62—C63	120.4 (3)
C11—C16—C15	121.1 (3)	C61—C62—H62A	119.8
C11—C16—H16A	119.4	C63—C62—H62A	119.8
C15—C16—H16A	119.4	C64—C63—C62	120.7 (3)
C22—C21—C26	118.4 (3)	C64—C63—H63A	119.6
C22—C21—P1	118.3 (2)	C62—C63—H63A	119.6
C26—C21—P1	123.2 (3)	C63—C64—C65	119.5 (3)
C23—C22—C21	121.5 (3)	C63—C64—H64A	120.2
C23—C22—H22A	119.3	C65—C64—H64A	120.2
C21—C22—H22A	119.3	C64—C65—C66	119.9 (3)
C22—C23—C24	119.1 (4)	C64—C65—H65A	120.1
C22—C23—H23A	120.5	C66—C65—H65A	120.1
C24—C23—H23A	120.5	C65—C66—C61	121.8 (3)
C25—C24—C23	120.2 (3)	C65—C66—H66A	119.1
C25—C24—H24A	119.9	C61—C66—H66A	119.1
